# Metabolic rewiring of the dendritic cell–T cell axis: tumour-derived barriers and therapeutic opportunities

**DOI:** 10.1038/s12276-026-01798-w

**Published:** 2026-08-06

**Authors:** Minkyu Cho, Minkyung Song

**Affiliations:** 1 Department of Biopharmaceutical Convergence, Sungkyunkwan University, Suwon, Republic of Korea; 2 Department of Integrative Biotechnology, Sungkyunkwan University, Suwon, Republic of Korea; 3 Personalize Cancer Immunotherapy Research Center, Sungkyunkwan University, Suwon, Republic of Korea

**Keywords:** Immunosurveillance, Cancer metabolism

## Abstract

The tumour microenvironment imposes severe metabolic constraints that reshape anti-tumour immunity across the cancer-immunity cycle. Rather than serving merely as passive byproducts of tumour growth, tumour-derived metabolites and nutrient imbalances act as potent metabolic checkpoints−stage-specific barriers that disrupt the functional progression of dendritic cells (DCs) and T cells from antigen presentation to effective tumour clearance. In this review, we propose a framework that overlays the cancer-immunity cycle with major metabolic checkpoints, including glucose and amino acid competition, acidosis and lipid overload, to clarify how distinct metabolic stresses create immune bottlenecks at different stages of the anti-tumour response. We then discuss how distinct tumour metabolic phenotypes, characterized by high glycolysis, amino acid dependency or lipid dysregulation, generate local environmental stresses that differentially reprogram DC function and T cell fitness. Particular emphasis is placed on the DC–T cell axis as a critical site where multiple metabolic defects converge, destabilizing antigen presentation, co-stimulation and immunological synapse function. We further survey emerging therapeutic strategies aimed at restoring the DC–T cell axis and effective anti-tumour immunity, ranging from small-molecule metabolic inhibitors to metabolically engineered adoptive cell therapies designed to function in hostile microenvironments. Finally, we highlight emerging technologies such as single-cell and spatial multi-omics, real-time metabolic imaging and microphysiological systems that can resolve the spatiotemporal heterogeneity of tumour immunometabolism and support more precise immunometabolic interventions.

## Tumour Metabolism: Disrupting Immune Cell Communication

The tumour microenvironment creates metabolic barriers that weaken anti-tumour immunity. This review explains how tumour-derived metabolites and nutrient imbalances act as metabolic checkpoints across the cancer-immunity cycle, disrupting dendritic cell antigen presentation, T cell activation and tumour killing. The authors organize these barriers around major tumour metabolic phenotypes, including high glycolysis, amino acid dependency and lipid dysregulation, and emphasize the dendritic cell–T cell interface as a vulnerable site where metabolic stress can destabilize co-stimulation and immunological synapse formation. The review also discusses therapeutic strategies and emerging technologies, such as single-cell and spatial multi-omics, organoids and microphysiological systems, that may help to identify and overcome metabolic bottlenecks to improve cancer immunotherapy.

This summary was initially drafted using artificial intelligence, then revised and fact-checked by the author.

## Introduction

The tumour microenvironment (TME) is a complex ecosystem comprising tumour cells, stromal cells, vascular components, fibroblasts and diverse immune-cell populations^1^. This dynamic milieu collectively forms a regulatory network that is crucial for tumour progression and immune evasion^1^. Beyond its cellular complexity, the TME exhibits pronounced spatial and metabolic heterogeneity, driven by aberrant vascular architecture and the high bioenergetic demands of tumour cells^1^. These factors create a hostile milieu characterized by severe hypoxia, nutrient scarcity and chronic acidosis^1,2^. Within this setting, the accumulation of metabolic stressors — such as reactive oxygen species (ROS), lactate and specific lipid species — does not merely reflect metabolic waste. Rather, these factors function as active metabolic checkpoints that impose bioenergetic and signalling constraints on infiltrating immune cells, thereby disrupting multiple stages of the cancer-immunity cycle (CIC), from antigen presentation and T cell activation to intratumoural function and tumour-cell killing^3^.

Within this metabolic landscape, the dendritic cell (DC)–T cell axis occupies a central position in determining the outcome of anti-tumour immunity. DCs, as professional antigen-presenting cells, are essential for capturing and processing tumour antigens, migrating to lymphoid tissues, and priming naïve or memory T cells to elicit antigen-specific effector responses^4^. However, DCs in the TME are highly sensitive to metabolic cues and can be readily reprogrammed from an immunostimulatory state toward dysfunctional or tolerogenic phenotypes, typically characterized by impaired antigen presentation, defective co-stimulation and altered cytokine production^4^. Concurrently, T cells exposed to nutrient depletion, immunosuppressive metabolites and acidic pH often undergo defective metabolic reprogramming, resulting in exhaustion, anergy or terminal differentiation rather than durable effector and memory function^5^. Although the CIC provides a stage-wise framework for anti-tumour immunity, many of these metabolically imposed defects converge at the DC–T cell interface, where antigen display, co-stimulation, signalling thresholds and effector execution are locally integrated. In this sense, the DC–T cell interface represents a critical metabolic hub through which tumour-induced perturbations are translated into immune failure.

This review specifically focuses on how metabolic pressures within the TME reshape DCs and T cell states and how these perturbations propagate across the CIC. We first define the concept of metabolic checkpoints and overlay them onto the CIC to clarify how stage-specific metabolic barriers disrupt immune progression. We then summarize major tumour metabolic phenotypes and the local environmental stresses they impose, before discussing how these conditions reprogram DC function, T cell fitness and, ultimately, the DC–T cell interface, where immune activation is locally executed or aborted. Finally, we discuss therapeutic strategies and emerging technologies — including single-cell and spatial multi-omics, organoid models and microfluidic-based microphysiological systems, that may resolve the spatiotemporal heterogeneity of tumour immunometabolism and support more precise immunometabolic interventions.

## The cancer-immunity cycle overlaid with metabolic checkpoints

**Fig. 1:**
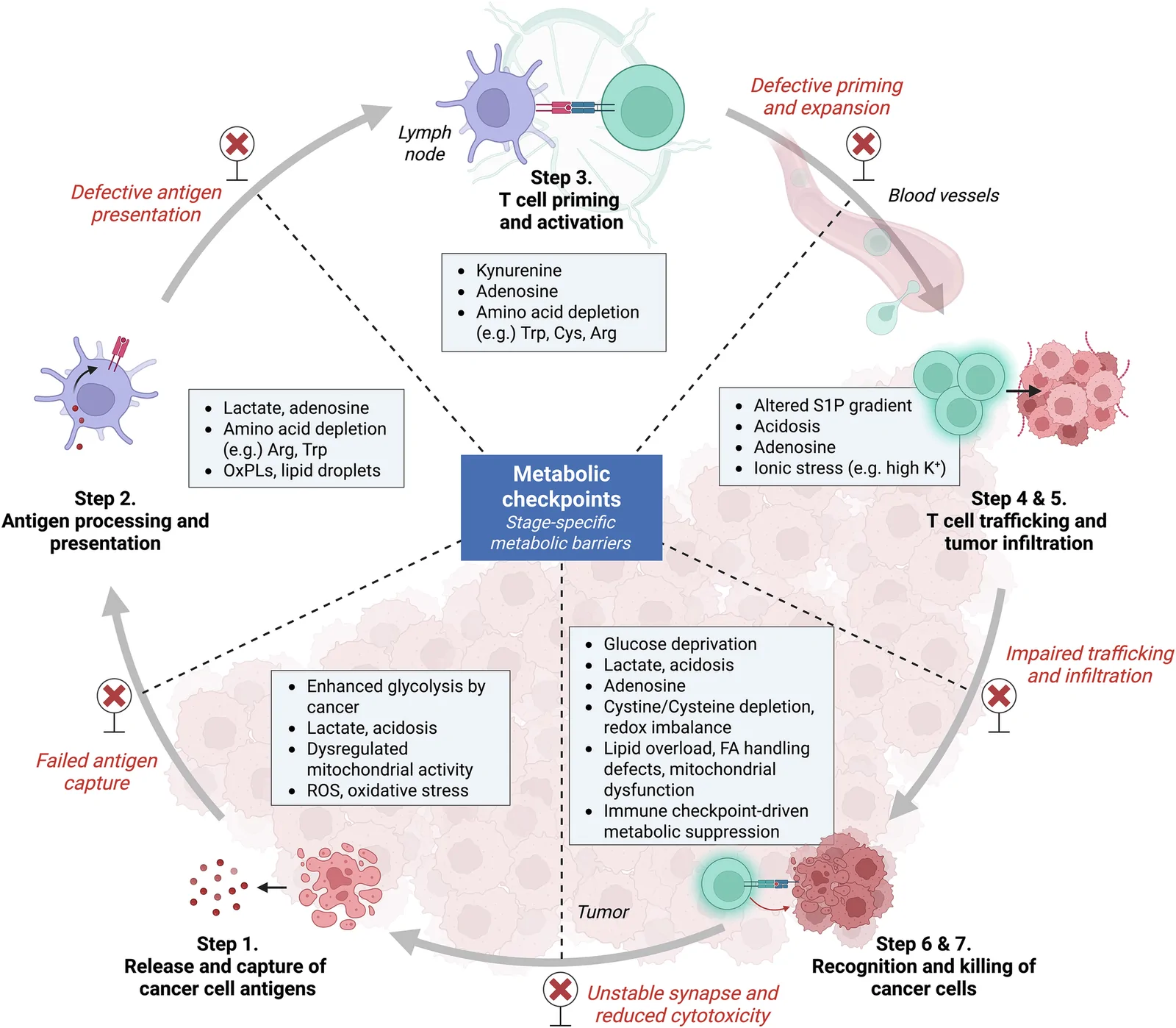
Stage-specific metabolic checkpoints in the cancer-immunity cycle. This conceptual framework illustrates how tumour-imposed metabolic alterations function as checkpoints across distinct stages of the cancer-immunity cycle (CIC). Rather than acting as uniform suppressive cues, individual metabolic stressors acquire stage-specific immunological consequences depending on where they intersect with dendritic-cell and T cell biology. During early CIC steps, these metabolic constraints limit the immunogenic release, capture, processing and presentation of tumour antigens by dendritic cells. At the priming phase, they restrain T cell activation and expansion in tumour-draining lymph nodes. During the effector phase, they further impede T cell trafficking, tumour infiltration, immunological synapse stability and cytotoxic function. Thus, metabolic checkpoints create discrete immune barriers that progressively uncouple antigen recognition from effective tumour clearance. This framework highlights how tumour metabolic phenotypes may impose distinct bottlenecks along the CIC and thereby reveal stage-specific vulnerabilities for therapeutic intervention. FA, fatty acid; ROS, reactive oxygen species. Schematic created with BioRender.com.

The CIC comprises sequential steps that collectively determine whether tumour-specific immune responses are initiated, expanded and executed^2^. In this framework, metabolic checkpoints are defined as metabolic barriers whose checkpoint-like effects become functionally decisive at particular stages of the CIC, thereby constraining effective anti-tumour immunity (Fig. 1). Importantly, some stressors — such as lactate, adenosine or amino acid depletion — may operate across multiple stages, but their functional consequences are manifested in a stage-dependent manner.

### Step 1. Release and capture of cancer-cell antigens: lactate and reactive oxygen species as early brakes on dendritic-cell immunogenicity

The CIC is initiated when stressed or dying tumour cells release antigens together with immunogenic signals that promote DC sensing and uptake^2^. This early step therefore depends not only on antigen availability, but also on the immunogenic quality of dying tumour cells and the functional competence of DCs. In the TME, enhanced glycolysis in cancer cells leads to excessive lactate production and extracellular acidification, whereas dysregulated mitochondrial activity generates high levels of ROS, increasing oxidative stress^6^. These changes function as early metabolic checkpoints: lactate dampens DC antigen uptake, maturation and responsiveness to danger signals, whereas excessive ROS damage lipids and proteins required for proper DC activation^6^. Together, these alterations reduce the immunogenicity of dying tumour cells and bias DCs towards dysfunctional or tolerogenic states.

### Step 2. Antigen processing and presentation: metabolite stress, amino acid depletion and oxidized lipids in dendritic-cell dysfunction

For effective antigen presentation, DCs coordinate glycolysis and oxidative phosphorylation to support antigen processing, peptide-MHC loading and co-stimulation^7^. In addition, glutamine, cysteine, arginine and tryptophan are essential for redox balance, differentiation and cytokine production^8^. In the TME, adenosine and lactate directly suppress DC metabolic activity and inflammatory signalling; likewise, the depletion of tryptophan and arginine — driven by enzymes such as indoleamine 2,3-dioxygenase (IDO)/tryptophan 2,3-dioxygenase (TDO) and arginase (ARG1) — act as a metabolic brake that reduces DC immunogenicity, MHC expression and IL-12 production^8–10^. Furthermore, the accumulation of oxidized lipids and lipid droplets interferes with antigen processing and presentation capacity^11,12^. Together, these checkpoints convert DCs from stimulatory antigen-presenting cells into dysfunctional cells that fail to effectively prime T cells.

### Step 3. T cell priming and activation: metabolite- and amino acid-driven barriers in tumour-draining lymph nodes

During T cell priming in lymphoid organs, TCR and co-stimulatory signals drive PI3K–AKT–mTOR activation to support the metabolic switch to glycolysis and enhanced mitochondrial activity^13^. Within tumour-draining lymph nodes, metabolically reprogrammed DCs can impair the quality of T cell priming by limiting co-stimulation and IL-12-dependent effector imprinting^14^. Suppressive amino acid and metabolite checkpoints such as IDO/TDO-kynurenine and adenosine restrain mTORC1-linked anabolic programs and enforce transcriptional programs associated with T cell anergy and exhaustion^9,15^. Furthermore, depletion of essential amino acids including arginine, tryptophan and cystine/cysteine limit protein synthesis and redox control, further compromising clonal expansion and effector differentiation of tumour-specific T cells^16,17^.

### Steps 4–5. T cell trafficking and tumour infiltration — S1P, acidosis, adenosine and ionic stress as motility checkpoints

Effective trafficking and infiltration require adequate ATP generation, intact cytoskeletal remodelling, and tightly regulated ionic fluxes^18,19^. In addition, sphingosine-1-phosphate (S1P) gradients contribute to vascular-to-tissue entry of effector T cells by tuning endothelial permissiveness and migratory responsiveness^20^. Within the TME, extracellular acidosis, adenosine accumulation and elevated extracellular potassium act as metabolic checkpoints that impair chemokine receptor signalling, cytoskeletal dynamics, and ion channel activity, thereby reducing T cell motility^21–24^. In metabolically altered tumours, disruption of the S1P axis may further impair transendothelial migration^25^, whereas local metabolic checkpoints impede subsequent interstitial motility within the TME, thereby limiting the ability of DC-primed effector T cells to reach and infiltrate tumour nests and disconnecting upstream priming from downstream tumour-cell killing.

### Steps 6–7. Recognition and killing of cancer cells: synapse-destabilizing and effector-limiting metabolic checkpoints

After infiltration, tumour-specific T cells must repeatedly recognize cognate peptide-MHC and assemble a stable immunological synapse, in which receptor clustering, adhesion, cytoskeletal remodelling and sustained signalling are integrated to enable cytotoxic activity^26,27^. Mitochondrial repositioning and local ATP supply support activation-linked signalling at the synapse, whereas physiological mitochondrial ROS contributes to antigen-driven T cell activation, rendering this phase highly sensitive to local metabolic stress^28–30^. Tumour-driven glucose restriction reduces the glycolytic reserve required for rapid TCR-proximal signalling and cytoskeletal remodelling, whereas lactate, acidosis and adenosine blunt activation-linked signalling and destabilize synapse assembly^9,31,32^. In parallel, limited cystine/cysteine availability and impaired glutathione-dependent redox buffering restrict activated T cell function and may compromise the cytoskeletal stability required for sustained signalling^33,34^.

Once recognition occurs, continued cytotoxicity further depends on glucose availability, amino acid-supported metabolic fitness and mitochondrial function for cytokine production and repeated killing^35–37^. Lipid overload, defective fatty acid handling and mitochondrial dysfunction accelerate terminal exhaustion, whereas signalling of checkpoint receptors such as PD-1 and CTLA-4 further suppresses glycolytic and mitochondrial fitness^38–40^. Together, these checkpoints shorten the cytotoxic lifespan of intratumoural T cells and limit durable tumour control.

Collectively, these metabolic checkpoints across the CIC progressively decouple antigen recognition from effective tumour clearance by imposing stage-relevant barriers on DC and T cell function. Rather than acting as passive consequences of tumour growth, these metabolic stresses create discrete immune bottlenecks that are shaped by tumour-intrinsic metabolic phenotypes and the local environmental conditions that they generate. This framework therefore provides explanatory value by linking distinct tumour-derived metabolic stresses to stage-specific immune bottlenecks, and predictive value by identifying which steps of the CIC may be most vulnerable to intervention in different metabolic tumour contexts. Understanding how high-glycolytic, amino acid-dependent, or lipid-dysregulated tumours reprogram DC function, T cell fitness and ultimately the DC–T cell axis is therefore essential for identifying vulnerable nodes for therapeutic intervention. In the following section, we examine how distinct tumour metabolic phenotypes generate local environmental stresses that differentially remodel anti-tumour immunity, with particular emphasis on the convergence of these defects at the level of antigen presentation, co-stimulation and immunological synapse function.

#### Core tumour metabolic phenotypes and their stage-specific impacts on the cancer-immunity cycle

Metabolic reprogramming within the TME is a hallmark of cancer that transcends histological types, collectively shaping the extracellular milieu into a hostile landscape for immune cells^41^. Rather than a uniform process, recent single-cell analyses have identified three core metabolic phenotypes — high-glycolysis, high-amino acid and high-lipid types — each associated with distinct molecular drivers and immune interactions^42,43^ (Table 1). These tumour-intrinsic programs do not merely meet the biosynthetic demands of cancer cells but function as active architects of the TME, installing potent metabolic checkpoints across the CIC.

**Table 1:** Core tumour metabolic phenotypes and their stage-specific impacts on the cancer-immunity cycle.

Metabolic phenotype	Representative tumour types and molecular drivers	Key regulators	Immune suppressive checkpoint mechanisms	Major CIC steps affected
High-glycolysis	NSCLC (KRAS mut/LKB1 loss), Luminal B Breast, GBM (EGFR amp/PTEN loss), CRC (KRAS mut)	GLUT1 (SLC2A1), HK2	Glucose depletion starves immune cells	Steps 2, 6 and 7
		LDHA, MCT1	Lactate/acidosis inhibits pH-sensitive glycolytic enzymes, DC maturation and T cell effector function	Steps 2, 6 and 7
		PKM2	PKM2-HIF1α axis upregulates PD-L1, reinforcing immune-metabolic suppression at the tumour–T cell interface	Steps 6 and 7
High-amino acid	PDAC, TNBC (c-MYC), ccRCC (BACH1/2), CRC	IDO1, TDO	Catabolizes tryptophan to kynurenine Activates AhR to drive T cell exhaustion	Steps 2 and 3
		SLC1A5, GLS1	Aggressive Gln consumption starves DCs and T cells of biosynthetic precursors	Steps 2, 3, 6 and 7
		ARG1, ARG2	Depletes Arg, leading to reduced CD3ζ expression and impaired TCR signalling	Steps 2, 3, 6 and 7
		SLC7A11 (xCT)	Limits cystine/cysteine availability and redox buffering, impairing DC competence and T cell proliferation/function	Steps 2, 3, 6 and 7
High-lipid	OvCa, HCC (SOX2), COAD, Glioma/GBM	CD36, FASN	Drives lipid overload and OxPLs production; OxPLs disrupt pMHC trafficking in DCs	Steps 2, 6 and 7
		COX-2	Secretes PGE_2_-rich signalling, tolerogenic myeloid programming and suppression of productive anti-tumour immunity	Steps 2, 6 and 7
		SREBPs, CPT1A	Supports lipid-rich, stress-adaptive tumour states, contributing to lipid overload and impaired intratumoural T cell fitness	Steps 6 and 7
		SPHK1	SPHK1/S1P axis promotes Treg accumulation and immunosuppressive cytokine signalling, thereby restricting CD8^+^ TIL infiltration and effector function	Steps 4, 5, 6 and 7

*ccRCC* clear-cell renal-cell carcinoma, *CIC* cancer-immunity cycle, *COAD* colon adenocarcinoma, *CRC* colorectal cancer, *DC* dendritic cell, *GBM* glioblastoma multiforme, *HCC* hepatocellular carcinoma, *NSCLC* non-small-cell lung cancer, *OvCa* ovarian cancer, *PDAC* pancreatic ductal adenocarcinoma, *TNBC* triple-negative breast cancer.

### High-glycolysis tumours: fuelling acidosis and competition

Driven by KRAS mutations, LKB1 or PTEN loss and EGFR amplification, high-glycolysis tumours — such as non-small-cell lung cancer (NSCLC), glioblastoma multiforme and colorectal cancer — implement an extreme Warburg effect, producing and exporting vast amounts of lactate that acidify the TME, even under oxygen-replete conditions^44–46^. Guided by the coordinated upregulation of glucose transporters such as GLUT1 and rate-limiting enzymes including hexokinase II (HK2), phosphofructokinase-1 (PFK-1), pyruvate kinase M2 (PKM2) and lactate dehydrogenase A (LDHA), these tumours support anabolic diversion of glycolytic intermediates while simultaneously depleting the local glucose pool^47^ (Table 1). Together, glucose competition and lactate-driven acidosis create metabolic constraints that can function as stage-specific metabolic checkpoints along the CIC, particularly by limiting optimal DC activation (Step 2) and weakening intratumoural T cell effector fitness (Steps 6–7)^35,48^. Within the acidic milieu, pH-sensitive glycolytic enzymes — specifically PFK-1 and glyceraldehyde-3-phosphate dehydrogenase (GAPDH) — and intrinsic signalling pathways mediated by mTORC1 or NFAT are functionally inhibited, thereby arresting the glycolytic flux necessary for immune-cell proliferation and effector cytokine production^49,50^. Notably, PKM2 functions beyond its canonical role in glycolysis by acting as a transcriptional co-activator for HIF-1α, which directly upregulates PD-L1 expression on tumour cells^51,52^. This PKM2–HIF-1α–PD-L1 axis installs a formidable immune-metabolic checkpoint at the tumour–T cell interface (Steps 6–7), suppressing T cell effector functions by inhibiting the PI3K/Akt/mTOR signalling pathway and further impairing glycolytic flux, which collectively promotes immune evasion in high-glycolysis tumours^38^.

### High-amino acid tumours: nutrient depletion and catabolite-driven tolerance

High-amino acid tumours are characterized by increased uptake and utilization of amino acids, as observed in metabolically aggressive tumours such as pancreatic ductal adenocarcinoma, c-MYC-driven triple-negative breast cancer, clear-cell renal-cell carcinoma, and subsets of colorectal cancer and NSCLC^53–56^. One key feature of this phenotype is glutamine addiction, driven by transporters such as SLC1A5 (ASCT2) and glutaminolysis enzymes including glutaminase 1/2 (GLS1/2)^57^. In tumours, glutamine supports anaplerosis, nucleotide synthesis, redox control and survival under oxidative stress^57^. In addition, many tumours downregulate argininosuccinate synthase 1 (ASS1) and become dependent on exogenous arginine, while upregulating CAT family transporters and arginase (ARG1) activity to support proliferation, polyamine synthesis, angiogenesis and nitric oxide-associated adaptation^57,58^. High-amino acid tumours also frequently enhance tryptophan catabolism through IDO1/TDO, generating kynurenine and downstream metabolites that contribute to de novo NAD⁺ synthesis, oxidative stress resistance and metastatic fitness^59^. In addition, cystine/cysteine metabolism supports tumour survival by sustaining glutathione production, ferroptosis resistance and mitochondrial adaptation through transporters such as SLC7A11/xCT^60,61^.

Although these amino acid programs support tumour growth through distinct biosynthetic and stress-adaptive mechanisms, their immunological consequences converge on a more limited set of checkpoint functions across the CIC^57^. Rather than acting through entirely separate immune pathways, high amino acid tumours primarily impose nutrient-deprivation checkpoints. Competition for glutamine, arginine and cystine/cysteine limits substrates required for DC metabolic fitness, inflammatory competence and antigen presentation, thereby weakening antigen presentation (Step 2) of the CIC^8,57,62^. The same nutrient restriction also constrains T cell biosynthetic and redox programs required for clonal expansion and effector commitment during priming (Step 3) and can further erode sustained intratumoural effector function during recognition and killing (Steps 6–7)^17^. In parallel, tryptophan catabolism through IDO1/TDO establishes a catabolite-driven tolerogenic checkpoint that reinforces dysfunctional priming and impaired effector differentiation, particularly at Steps 2–3^57,63^. The accumulation of kynurenine further activates aryl hydrocarbon receptor (AhR)-linked tolerogenic programs, suppressing DC immunogenicity and promoting anergy-like T cell differentiation^59^.

#### High-lipid tumours: lipid overload and inflammatory lipid-mediated suppression

High-lipid tumours are defined by enhanced lipid acquisition, storage and utilization, a pattern observed in metabolically adaptive malignancies such as ovarian cancer, SOX2-overexpressing hepatocellular carcinoma, colon adenocarcinoma and glioma/glioblastoma^64–67^. This state is sustained by coordinated upregulation of fatty acid translocase CD36 and fatty acid transport proteins (SLC27 family), together with activation of de novo lipogenic and fatty acid oxidation pathways controlled by SREBPs and FASN, CPT1A, HSL and MAGL^66,68^. These adaptations provide more than a bioenergetic advantage: they enable membrane remodelling, resistance to oxidative stress and persistence under hypoxic or nutrient-limited conditions, while also enriching the tumour milieu with bioactive lipid species^66,69^. As a result, high-lipid tumours generate a microenvironment that is not only lipid rich but also functionally skewed towards immune dysfunction through lipid mediators^40,70,71^.

From an immunological perspective, this phenotype most prominently affects the CIC by altering how immune cells encounter, process and respond to lipid excess. In DCs, excess lipids and oxidized lipid species interfere with antigen processing and presentation, thereby weakening antigen presentation competence at Step 2^11^. In parallel, T cells exposed to chronic lipid excess undergo abnormal fatty acid handling and lipid peroxidation, which increase mitochondrial stress and promote dysfunction, ultimately reducing cytotoxic fitness during recognition and killing at Steps 6–7^40,72^. Thus, the immunosuppressive effects of high-lipid tumours arise not primarily from nutrient deprivation but from chronic lipid overload and aberrant lipid exposure that distorts immune-cell function. An additional layer of suppression arises from lipid-derived signalling molecules such as lysophospholipids and prostaglandins. PGE_2_-rich conditions promote tolerogenic myeloid programming and dampen productive anti-tumour immunity, whereas dysregulated SphK1/S1P signalling may impair effector T cell infiltration during Steps 4–5 while favouring Treg accumulation and immunosuppressive remodelling of the TME^70,73^. In this way, high-lipid tumours preferentially establish bottlenecks at antigen presentation, tissue entry and sustained intratumoural effector activity across the CIC.

## Metabolic rewiring of dendritic cells as an early checkpoint in the cancer-immune cycle

### Metabolic fundamentals of dendritic cell activation

**Fig. 2:**
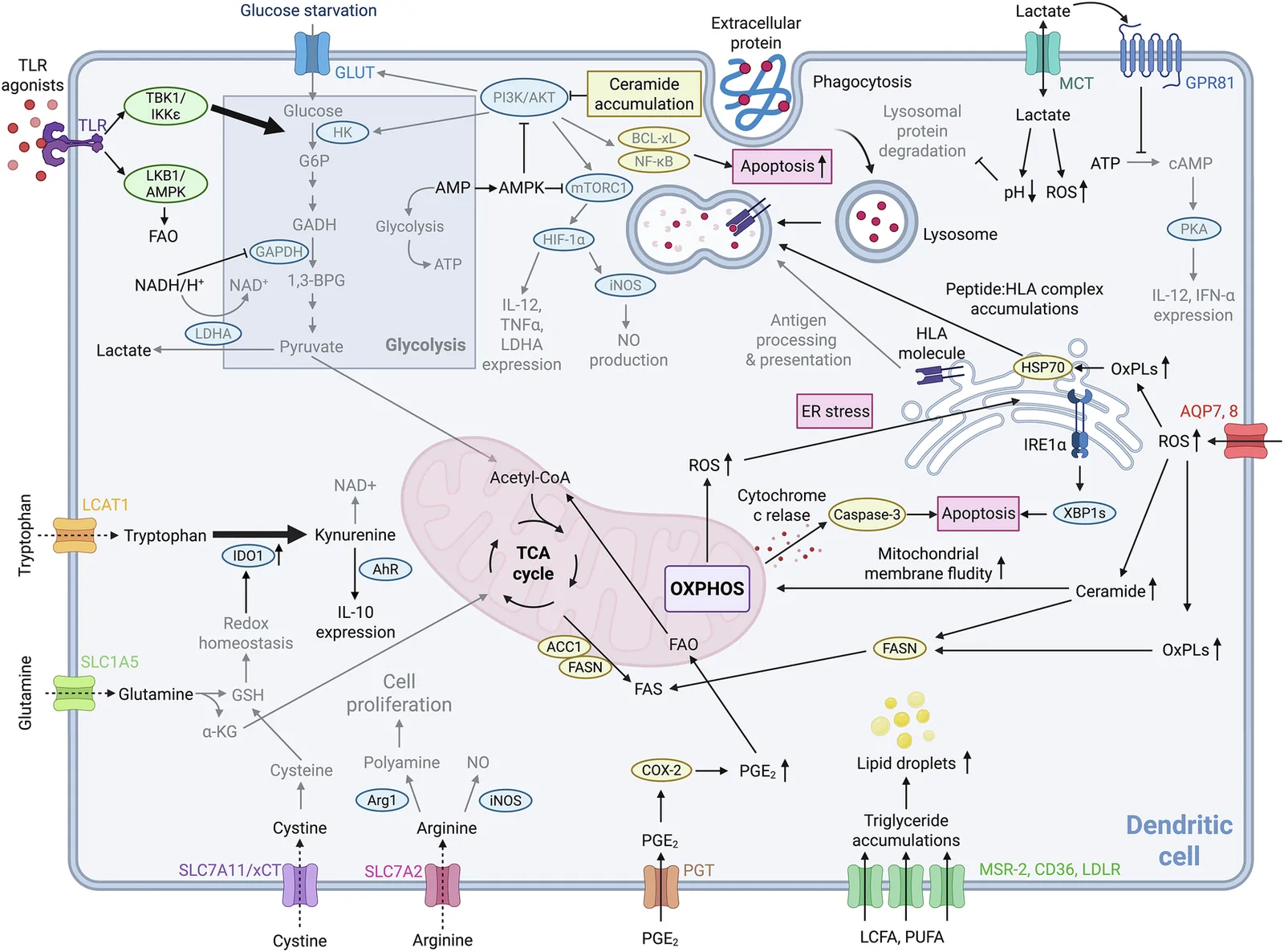
Metabolic rewiring and functional suppression of dendritic cells in the tumour microenvironment. This schematic illustrates how tumour-imposed metabolic stress rewires central metabolic pathways in dendritic cells and reshapes their fate. Metabolic reprogramming: pathways actively driving dysfunction or stress responses in the tumour microenvironment are depicted in black, whereas homeostatic pathways (such as glycolysis) that are suppressed are shown in grey. Tumour microenvironment-derived factors — including glucose starvation, lactate accumulation and lipid overload — inhibit central glycolysis (e.g. via GAPDH suppression) and divert metabolism toward fatty acid oxidation (FAO) and mitochondrial oxidative phosphorylation (OXPHOS). Lipid and amino acid dysregulation: Upregulation of lipid transporters (e.g. CD36, MSR-2) leads to the accumulation of triglycerides (lipid droplets) and ceramides. Concurrently, amino acid metabolism is altered via IDO1-mediated tryptophan catabolism and Arg1-mediated arginine depletion, contributing to immune tolerance. Functional consequences: these metabolic shifts trigger endoplasmic reticulum stress (via the IRE1α–XBP1s axis) and reactive oxygen species (ROS) generation, ultimately leading to Caspase-3-dependent apoptosis. Crucially, the accumulation of oxidized phospholipids (OxPLs) disrupts HSP70 function, blocking the trafficking of Peptide–HLA complexes to the cell surface and thereby abrogating antigen presentation. Additionally, lactate signalling (via GPR81) and metabolic stress suppress pro-inflammatory cytokines (IL-12, TNF-α) while promoting IL-10 expression. Schematic created with BioRender.com.

DC activation is not merely a transcriptional change but a coordinated metabolic reprogramming process designed to meet the rigorous bioenergetic and biosynthetic demands of antigen uptake, processing, presentation, cytokine production and migration to lymphoid tissues^7,74^. Upon pattern recognition receptor (PRR) stimulation, DCs undergo a rapid metabolic shift from oxidative phosphorylation (OXPHOS) towards aerobic glycolysis, a process primarily driven by the TBK1–IKKε–Akt signalling axis^7,75^ (Fig. 2). This glycolytic commitment is reinforced by the upregulation of the rate-limiting enzyme HK2, which accelerates glycolytic flux^7^. Concurrently, activated DCs produce nitric oxide via inducible nitric oxide synthase (iNOS), which suppresses mitochondrial respiration by inhibiting Complex IV of the electron transport chain (ETC), thereby locking DCs into a glycolytic state^76^. This “Warburg-like” metabolic program serves two critical functions: first, it provides the rapid ATP burst necessary for cytoskeletal remodelling, antigen uptake and migration towards lymphoid tissues; second, it diverts glycolytic intermediates into the pentose phosphate pathway and de novo fatty acid synthesis^7,75^. These anabolic pathways support NADPH production, membrane biogenesis and the massive expansion of the endoplasmic reticulum (ER) and Golgi apparatus required for high-volume synthesis and transport of cytokines and MHC molecules^7,75^. Thus, metabolic rewiring is integral to the ability of DCs to convert tumour-derived antigenic material into productive T cell priming signals.

Although this glycolytic burst is a hallmark of DC activation, specific DC subsets exhibit distinct intrinsic metabolic dependencies tailored to their immunological functions. Conventional DC type 1 (cDC1s), which specialize in antigen cross-presentation to CD8^+^ T cells, maintain substantial mitochondrial flexibility^77,78^. Even after activation, cDC1s engage the LKB1–AMPK axis and fatty acid oxidation (FAO) to support the bioenergetic demands of cross-presentation and to manage ROS^4,79^. In contrast, cDC2s, which are essential for CD4^+^ T cell priming, rely more heavily on glycolysis and are functionally regulated by ROS signalling and metabolic cues such as lipid accumulation^7,79^. This intrinsic metabolic heterogeneity renders specific DC subsets differentially vulnerable to metabolic constraints in the TME; for instance, the dependence of cDC1s on mitochondrial fitness may make them particularly sensitive to lipid peroxidation and hypoxic stress^77,78,80^.

### Glucose deprivation and lactate accumulation: a dual metabolic blockade on dendritic cell maturation

The high glycolytic activity of tumour cells creates a glucose-scarce and lactate-rich environment, imposing a dual metabolic blockade on infiltrating DCs (Fig. 2). Glucose restriction directly dampens mTORC1 activity and prevents HIF-1α stabilization in DCs^74^. Given that HIF-1α acts as a critical transcriptional regulator for both glycolytic enzymes and inflammatory cytokine programs, its suppression compromises DC maturation and immunogenicity^74,75^. This defect is phenotypically characterized by reduced expression of co-stimulatory molecules such as CD80 and CD86, and MHC class II, alongside impaired production of IL-12, a cytokine critical for effective T cell priming and Th1 polarization^7,81,82^.

Tumour-derived lactate further suppresses DC function beyond its role as a metabolic byproduct^83,84^. Mechanistically, high intracellular lactate concentrations alter the NAD^+^/NADH ratio and inhibit GAPDH activity, thereby effectively stalling glycolytic flux and limiting energy production in DCs^82,83,85^. In addition, tumour-derived lactate and acidosis impair T cell priming by disrupting DC antigen processing and trafficking pathways, as shown by lactate-conditioned DCs with defective cross-presentation and tumour-conditioned DCs with reduced SNARE VAMP3, a regulator of endosomal trafficking required for tumour antigen cross-presentation^86,87^.

Furthermore, chronic exposure to lactate promotes DCs into a tolerogenic state, mediated either through direct metabolic inhibition or via signalling through the lactate receptor GPR81^77,88^. This signalling axis skews the cytokine profile towards immunosuppression — favouring IL-10 and TGF-β secretion over IL-12 — and enhances apoptosis within DC clusters^7,74,88^. Collectively, glucose restriction and lactate accumulation function as early metabolic checkpoints that converge to severely compromise DC maturation, antigen presentation and downstream T cell priming, ultimately driving tumour immune escape and attenuating responsiveness to immune-checkpoint inhibitors^4,7,77^.

### Amino acid checkpoints: nutrient sensing and tolerogenic programming

DCs sense amino acid availability not only as a source of nutrients, but also as a determinant of differentiation, stress adaptation and functional competence^8^. Within the TME, key amino acids — including arginine, tryptophan and cystine — are depleted through consumption by tumour cells and enzymatic degradation by myeloid-derived suppressor cells (MDSCs), tolerogenic DCs and tumour-associated macrophages^77,89^. These restrictions trigger stress-sensing pathways that convert DCs from immunogenic antigen-presenting cells into tolerogenic or dysfunctional cells^8,90^ (Fig. 2).

Arginine depletion exerts a direct translational blockade on DC function. High expression of ARG1 and iNOS in tumour-infiltrating myeloid cells rapidly consumes extracellular arginine^91,92^. As arginine is a rate-limiting substrate for protein synthesis, its scarcity diminishes the translation of high-turnover proteins essential for DC activation, including MHC and co-stimulatory molecules, and impairs cytokine secretion, thereby stalling the initiation of productive T cell responses^91,92^.

Tryptophan metabolism constitutes a self-reinforcing tolerogenic loop. DCs within the TME frequently express high levels of IDO1, which converts tryptophan into kynurenine^43^. Local tryptophan depletion triggers the metabolic stress-sensor GCN2, whereas kynurenine acts as an oncometabolite that activates the aryl hydrocarbon receptor (AhR)^8,93^. This IDO1–kynurenine–AhR axis is particularly potent in cDC2s, driving a regulatory phenotype characterized by IL-10 secretion and suppression of T cell proliferation^8,69,70^.

The glutamine–cystine–glutathione (GSH) axis further governs both DC survival and antigen-processing capacity^94^. DCs convert imported glutamine into glutamate via glutaminase, and this glutamate further fuels the tricarboxylic acid (TCA) cycle as α-ketoglutarate as well as serving as the obligate counter-ion for the xCT antiporter, which imports extracellular cystine, the rate-limiting precursor for GSH synthesis^8,95^. In the cystine-depleted TME, this transport mechanism stalls, resulting in reduced intracellular GSH^74,94^. This creates a dual bottleneck: first, the lower level of GSH compromises glutathione peroxidase 4 (GPX4) activity, rendering DCs highly susceptible to ferroptosis, an iron-dependent form of cell death; second, impaired redox buffering disrupts the reduction of disulfide bonds in antigens within lysosomes, thereby preventing the antigen unfolding and proteolytic processing required for effective T cell priming^8,94,96^.

### Lipid dysregulation: lipotoxicity and antigen-trafficking defects

Disrupted lipid metabolism represents another major immunosuppressive checkpoint that fundamentally compromises DC fitness in the TME (Fig. 2). As direct targets of lipid metabolites released by tumour cells, MDSCs and tumour-associated macrophages, DCs undergo pathological lipid reprogramming and acquire a “lipid-laden” phenotype characterized by defective membrane dynamics, antigen processing and maturation, ultimately leading to the induction of T cell tolerance rather than activation^57,74^.

The accumulation of lipids is driven by active uptake mechanisms. DCs sequester tumour-derived long-chain fatty acids and polyunsaturated fatty acids into cytosolic lipid droplets, a process mediated in part by upregulating the scavenger receptor MSR1 (SR-A)^97^. This accumulation is further exacerbated by a pathogenic feed-forward loop involving PGE₂^97,98^. PGE₂ has context-dependent effects on DCs, as transient PGE₂ signalling can support DC migration, maturation and inflammatory activation, whereas chronic exposure in the TME promotes suppressive outcomes^99^. High concentrations of PGE₂ induce COX-2 expression in DCs, further amplifying endogenous PGE₂ production and enhancing FAO; this metabolic shift paradoxically accelerates lipid accumulation and reinforces the suppression of antigen presentation^98^.

A critical consequence of this lipid burden is the physical blockade of antigen trafficking. Oxidized phospholipids, generated under conditions of elevated ROS and myeloperoxidase activity from MDSCs, accumulate within DC lipid bodies^11,77^. These reactive lipids form covalent adducts with heat shock protein 70 (HSP70), a chaperone essential for the transport of peptide–MHC complexes^11^. This biochemical modification results in intracellular retention of pMHC within late endosomes/lysosomes, preventing their translocation to the cell surface and thereby restraining the ability of DCs to prime CD8^+^ T cells^100^.

Beyond antigen-trafficking defects, lipid dysregulation also induces lipotoxicity and apoptosis via ceramide accumulation^101^. Inflammatory mediators in the TME enhance sphingomyelinase activity in DCs, leading to a sharp increase in toxic C16 and C24 ceramides^102,103^. This ceramide buildup suppresses the PI3K–Akt signalling axis, reducing downstream NF-κB and Bcl-xL expression, and thereby diminishes DC survival^102^. Furthermore, ceramides can permeabilize the mitochondrial outer membrane, triggering cytochrome c release and caspase-3 activation, which directly induces apoptosis^102,104^. Collectively, these lipid-driven metabolic checkpoints impair antigen-trafficking and T cell priming capacity, while also shaping DC adaptation to metabolic stress and compromising DC survival within the TME, thereby amplifying tumour-associated immunosuppression.

## Metabolic checkpoints governing T cell activation, trafficking and exhaustion

### Metabolism as a central regulator of T cell fate

**Fig. 3:**
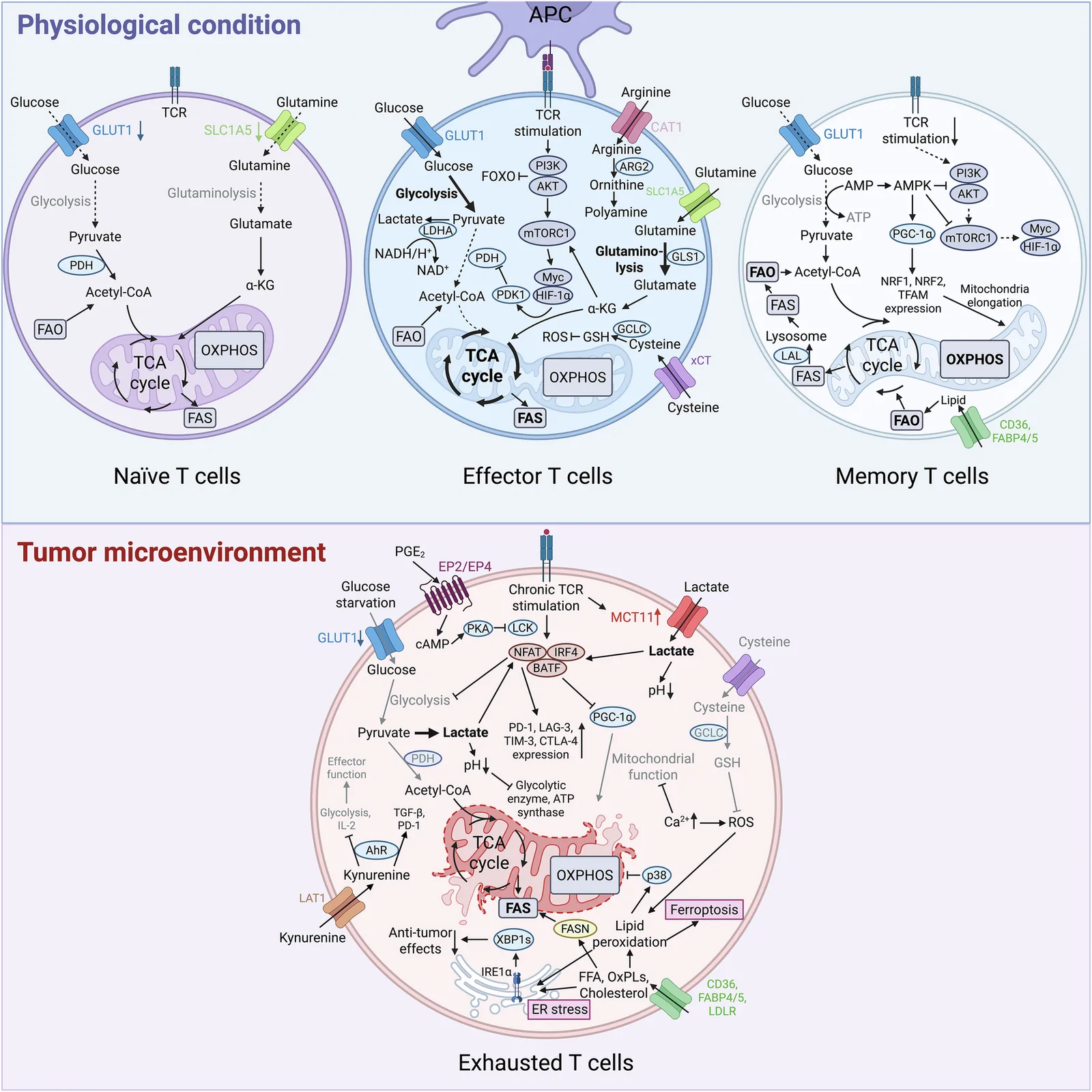
Distinct metabolic programming of T cell subsets under physiological conditions versus the tumour microenvironment. This schematic compares the metabolic profiles and regulatory networks of T cells during differentiation (upper panel) and exhaustion (lower panel). Physiological condition (upper panel): naïve T cells maintain metabolic quiescence with low nutrient transporter expression (low GLUT1), relying on mitochondrial oxidative phosphorylation (OXPHOS) and fatty acid oxidation (FAO). Upon activation, effector T cells undergo a metabolic switch mediated by the PI3K-Akt-mTORC1 and Myc-HIF-1α axes, upregulating glycolysis, glutaminolysis (GLS1), arginine uptake (CAT1) and fatty acid synthesis (FAS) to support rapid proliferation. Memory T cells revert to a catabolic state governed by the AMPK–PGC-1α axis, promoting mitochondrial biogenesis (NRF1, TFAM), elongation and reliance on FAO and OXPHOS for long-term survival. Tumour microenvironment (lower panel): exhausted T cells exhibit profound metabolic dysfunction driven by chronic stress. Glucose starvation and lactate influx (via MCT11) lower intracellular pH and suppress mitochondrial function. PGE_2_ activates cAMP-PKA signalling to inhibit LCK, whereas kynurenine activates AhR to upregulate PD-1. Chronic TCR stimulation activates NFAT, IRF4 and BATF, which drive the expression of immune checkpoints (PD-1, LAG-3, TIM-3, CTLA-4) and suppress PGC-1α. Accumulation of free fatty acid (FFA), cholesterol and oxidized lipids triggers ER stress (IRE1α**–**XBP1s axis), leading to lipid peroxidation and ferroptosis. Collectively, these alterations impair bioenergetics and anti-tumour immunity. Schematic created with BioRender.com.

T cells, as key components of the adaptive immune system, are supported by precisely orchestrated metabolic programs^105^. T cell metabolism provides the energy and biosynthetic resources needed for survival, proliferation, differentiation and immune function, undergoing marked remodelling as cells transition from the naïve to activated and effector states^106^. These metabolic changes are the major regulatory mechanisms determining T cell growth and functional characteristics, adjusted to the specific metabolic demands of various subtypes such as naïve, effector and memory T cells^105–107^ (Fig. 3). This fine-tuned metabolic control is therefore central to shaping T cell differentiation pathways and functional diversity^38,105,108^.

### Naïve T cells: metabolic quiescence and homeostasis

Naïve T cells are metabolically quiescent, relying on low levels of OXPHOS and FAO for energy production^105,109^ (Fig. 3). GLUT1 expression is low, and the activity of glycolytic enzymes such as HK2 and phosphofructokinase (PFKL) is minimized^105,109^. Glucose is primarily converted to acetyl-CoA via the pyruvate dehydrogenase (PDH) complex and enters the TCA cycle, generating NADH/FADH_2_, for efficient ATP production via the ETC^105,108^. Glutamine uptake is restricted owing to low ASCT2 expression, and only basal levels are converted to glutamate and α-KG via GLS1 to support TCA cycle anaplerosis^105^. Arginine, tryptophan and cysteine are minimally utilized, and GSH production remains at basal levels^106,110^. PPAR signalling sustains carnitine palmitoyltransferase 1α (CPT1 α) expression, enabling extracellular fatty acids to enter mitochondria via the carnitine shuttle for FAO, whereas de novo fatty acid synthesis remains minimal^105,110^.

### T cell activation and effector differentiation: glycolysis and glutaminolysis for anabolic growth

Upon TCR and CD28 stimulation, T cells undergo a rapid glycolytic switch that supports their transition from a quiescent state to activated and effector states^111^ (Fig. 3). TCR signalling activates LCK, ZAP-70 and LAT, recruiting PI3K to the membrane^105,106,108^. Activated PI3K–AKT signalling strongly activates mTORC1 while inhibiting the FOXO transcription factor^105,106,108^. The mTOR–Myc–HIF-1α axis increases the expression of glycolytic proteins such as GLUT1 and HK2^112^. Specifically, HIF-1α induces PDK1, which phosphorylates and inactivates pyruvate dehydrogenase, thereby blocking pyruvate entry into the TCA cycle. Instead, pyruvate is reduced to lactate by LDHA, allowing for NAD^+^ regeneration and sustained glycolysis^13,105,106^.

Glutaminolysis via SLC1A5 and GLS1 becomes active to support effector differentiation, with glutamine directly activating mTORC1 and supporting TCA-cycle anaplerosis^13^. Arginine is taken up via cationic amino acid transporter 1 (CAT1) and converted to ornithine by ARG2 for polyamine synthesis, which is essential for CD4^+^ T cell differentiation and DNA synthesis^107^. Cysteine promotes GSH synthesis via the glutamate–cysteine ligase catalytic subunit (GCLC) enzyme, helping to eliminate ROS generated during this intense metabolic reprogramming^13^. Thus, the transition from OXPHOS-dominant metabolism to aerobic glycolysis acts as a critical metabolic priming step that allows rapid ATP generation and provides biosynthetic precursors required for proliferation, cytokine production and the subsequent execution of effector function^105,109^.

### Memory T cells: fatty acid oxidation- and oxidative phosphorylation-centred metabolic flexibility and longevity

Following antigen clearance, reduced antigen stimulation and inflammation drive effector T cells towards memory states, accompanied by a metabolic shift from glycolysis back to FAO- and OXPHOS-centred metabolism^109,111^ (Fig. 3). AMPK activation and MAPK-dependent suppression of mTOR reduce glycolytic drive^108,113^. Attenuated TCR stimulation dampens the PI3K–AKT–mTORC1–Myc–HIF-1α axis, decreasing expression of glycolytic enzymes and inhibiting glycolytic flux^105,106,113^. Activated AMPK enhances signalling through PGC-1α, stimulating mitochondrial biogenesis by activating transcription factors NRF1, NRF2 and TFAM^114^. This remodels mitochondria into elongated organelles with dense cristae and enhanced OXPHOS capacity^106,115^. PPAR signalling promotes increased CPT1α expression, further supporting FAO^108,115^. Sustained OXPHOS and FAO are crucial for long-term survival and rapid recall responses characteristic of memory T cells^108^.

Although memory T cell subsets (Tcm, Tem, Trm) generally depend on mitochondrial metabolism for persistence and recall responses, they differ in how they acquire and use metabolic substrates^116^. Central memory T cells (Tcm) engage in a unique “futile cycle” that converts glucose into fatty acids, which are then oxidized via FAO^116,117^. Tcm cells exhibit lower lipid uptake than effector T cells (Teff) or tissue-resident memory T cells (Trm) and can persist in lipid-poor environments^123^. By contrast, Trm cells actively take up exogenous fatty acids from their tissue microenvironment, supported by high expression of lipid transport proteins such as FABP4/5 and CD36^118^. Effector memory T cells (Tem) rely more equally on glycolysis and OXPHOS and exhibit greater substrate flexibility than Tcm and Trm^108,118^.

### T cell intrinsic metabolic checkpoints driving exhaustion in the tumour microenvironment

Within the TME, the convergence of persistent antigenic stimulation and inhibitory signalling, and severe nutrient competition — driven by tumour cells and MDSCs — leads to a state of chronic metabolic stress^1,119^. This adverse environment drives T cells towards functional decline and terminal exhaustion, a state defined by profound defects in bioenergetics, cytokine secretion and cytotoxic functions^106,120^ (Fig. 3). The driver of this exhaustion is a distinct transcriptional and metabolic reprogramming triggered by chronic antigen exposure^109^. Continuous TCR stimulation sustains the activation of transcription factors such as NFAT, IRF4 and BATF^109,111,121^. Notably, in the context of exhaustion, NFAT often acts independently of AP-1; this uncoupled signalling drives a maladaptive transcriptional program that directly binds metabolic genes to suppress glycolysis and inhibit mTOR signalling^105,120^. Concurrently, the repression of PGC-1α transcription leads to severe mitochondrial dysfunction rather than a healthy transition to oxidative metabolism^121^.

Physiologically, this results in a state of “metabolic insufficiency”. Exhausted T cells exhibit reduced GLUT1 expression and glucose uptake, along with compromised mitochondrial fitness characterized by depolarized membrane potential and diminished activity of ETC complexes I–IV and ATP synthase^111,122^. These changes severely limit the cell’s spare respiratory capacity, rendering T cells vulnerable to energetic failure under metabolic stress^107,109,119^.

Furthermore, persistent TCR stimulation also elevates intracellular Ca²⁺ levels, which accelerates mitochondrial ROS production^123^. This oxidative stress creates a feed-forward loop that further activates NFAT^121,123^. In CD8⁺ T cells, this metabolic–transcriptional axis ultimately culminates in the direct binding of NFAT to the promoter regions of immune-checkpoint genes such as *LAG3*, *PDCD1* (PD-1), *HAVCR2* (TIM3) and *CTLA4*, thereby functioning as a key regulator that reinforces immune suppression^111,121,124^.

### Extrinsic metabolic checkpoints in the tumour microenvironment

Beyond intrinsic transcriptional rewiring, the TME imposes harsh extrinsic metabolic checkpoints that actively suppress T cell function (Fig. 3). High concentrations of lactate in the TME enter T cells via MCT1, lowering cytosolic pH^85^. This acidification inhibits pH-sensitive glycolytic enzymes, such as PFK, thereby reducing ATP production and halting cell proliferation^13^. Furthermore, lactate influx during chronic TCR stimulation modulates HIF-1α and NFAT activation to suppress IFNγ and IL-2 secretion while increasing PD-1, CTLA-4 and LAG3 expression, thereby effectively dampening T cell activation^124,125^.

Amino acid metabolism constitutes another formidable barrier. Tryptophan depletion and its enzymatic conversion to kynurenine by MDSCs and tumours represents a major metabolic checkpoint^59^. Kynurenine is readily taken up by tumour-infiltrating T cells, where it acts as a ligand for the AhR, leading to transcriptional programs that suppress effector function and promote the secretion of immunosuppressive cytokines such as IL-10 and TGFβ^59,126,127^. In AhR-expressing intratumoural CD8^+^ T cells, active kynurenine transport activates AhR-dependent transcription of *PDCD1*, whereas IL-2–STAT5-driven TPH1 expression promotes tryptophan catabolism to 5-hydroxytryptophan (5-HTP), further enabling AhR-mediated induction of exhaustion-associated genes such as *PDCD1*, *LAG3*, *ENTPD1* (CD39) and *HAVCR2*^128,129^. Thus, the kynurenine–AhR axis links tryptophan catabolism and kynurenine accumulation to exhaustion-associated dysfunction in tumour-infiltrating T cells^59,126,127,130^.

The lipid-rich TME further exacerbates T cell exhaustion or effector dysfunction through receptor-mediated signalling, lipid peroxidation, and organelle stress. Notably, PGE₂ activates cAMP–PKA signalling via EP2 and EP4 receptors, inhibiting LCK and thereby attenuating proximal TCR signalling^131^. Acting as a potent local immunosuppressor, this tumour-derived PGE₂ selectively targets TCF1^+^ stem-like TILs by antagonizing IL-2 responsiveness and mTORC1 activity, thereby restricting their intratumoural expansion and effector differentiation^132^. Consequently, IL-2 and IFNγ production decreases, T cell proliferation is reduced and recovery of effector and memory functions is impaired^131^. Uptake of free fatty acid and oxidized phospholipids via CD36 induces lipid peroxidation, augments ferroptosis and promotes p38 kinase-mediated exhaustion profiles while suppressing mitochondrial metabolism and OXPHOS^121^. In addition to these exhaustion-promoting mechanisms, dysregulated sphingolipid signalling can reshape T cell positioning and immune suppression within the TME. A recent pan-cancer integrative analysis identified SPHK1 as a potential metabolic checkpoint associated with a poor prognosis, altered immune-cell infiltration and increased immune-checkpoint expression across multiple cancers^133^. As SPHK1 generates S1P, tumour-associated SPHK1/S1P gradient may distort local trafficking cues and promote an immunosuppressive TME that limits productive cytotoxic T cell infiltration and effector activity^133^. Finally, cholesterol accumulation in CD8^+^ T cells drives exhaustion via the ER stress response^134^. When cholesterol enters T cells, it upregulates the ER stress-related transcription factor XBP1 and PD-1 expression^121^. Subsequently, PD-1 engagement alters mitochondrial and lipid metabolism^38^. In ovarian cancer, XBP1 activation is associated with reduced glucose and glutamine uptake, leading to impaired mitochondrial fitness and diminished antitumour activity^121,135^. Such stress conditions in CD8^+^ T cells lead to the suppression of transgelin-2, which disrupt cytoskeletal organization and fatty acid uptake via FABP5, thereby dampening mitochondrial activity and cytotoxic capacity^136^.

Collectively, T cells undergo dynamic metabolic reprogramming throughout their lifespan: from naïve T cells relying on energy-efficient oxidative metabolism, to effector T cells utilizing enhanced glycolysis and glutaminolysis, to memory T cells depending on FAO and OXPHOS, and finally to exhausted T cells undergoing metabolic collapse in the TME.

## The dendritic cell–T cell interface: synapse-centric metabolic crosstalk

The immunological synapse (IS) is a highly organized interface between DCs and T cells that coordinates antigen recognition, co-stimulation, adhesion and polarized signalling^27^. Beyond serving as a structural contact site, the IS functions as a discrete metabolic unit: a spatially confined platform in which local ATP supply, Ca^2+^ buffering, lipid raft organization, vesicle trafficking and cytoskeletal remodelling are coordinated independently of global cellular metabolic reprogramming^27,137,138^. This distinction is important because TME-derived metabolic stressors can selectively disrupt synapse-localized processes even when global activation programs are only partially affected^137^.

### Structural dynamics and local bioenergetics at the immunological synapse

**Fig. 4:**
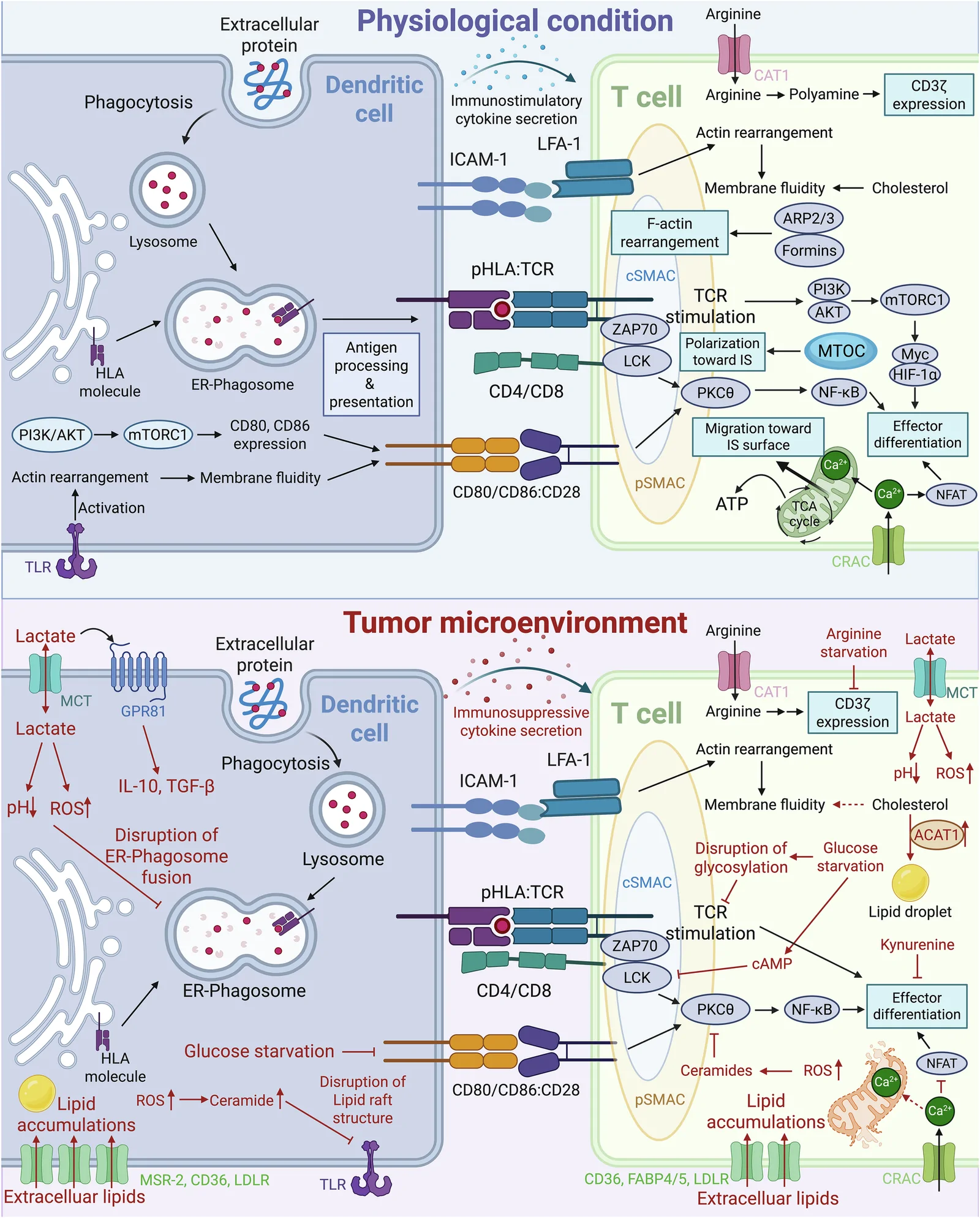
Metabolic and signalling architecture of the immunological synapse under physiological conditions versus the tumour microenvironment. This schematic contrasts the structural integrity and signalling efficacy of the dendritic cell (DC)–T cell synapse in health versus cancer. Physiological condition (upper panel): successful synapse formation relies on coordinated metabolic and cytoskeletal dynamics. DCs efficiently process antigens (ER-phagosome fusion) and upregulate co-stimulatory molecules (CD80/86) via PI3K-Akt-mTORC1 signalling. T cells exhibit robust actin rearrangement (mediated by ARP2/3 and formins) to stabilize the synapse (ICAM-1–LFA-1). Metabolic support is provided by arginine uptake (via CAT1) for CD3ζ expression, and mitochondrial ATP production. Calcium influx (Ca^2+^) and PKCθ recruitment drive NF-κB and NFAT signalling, culminating in effector differentiation. Tumour microenvironment (lower panel): synaptic dysfunction is driven by metabolic checkpoints. DC defects: lactate uptake and GPR81 signalling disrupt ER–phagosome fusion and induce suppressive cytokines (IL-10, TGFβ). Lipid accumulation (via MSR-2, CD36) generates ceramides, which disrupt lipid rafts necessary for antigen presentation. Glucose starvation dampens CD80/86 expression. T cell defects: arginine starvation leads to CD3ζ loss. Glucose deprivation alters receptor glycosylation. Lipid overload (via CD36, FABP4/5) and ACAT1-mediated cholesterol esterification promote lipid droplet formation. Accumulated reactive oxygen species (ROS) and ceramides inhibit PKCθ, whereas cAMP and kynurenine block downstream signalling (LCK, effector differentiation). Consequently, the synapse is physically unstable and signalling incompetent. Red arrows denote inhibitory pathways; blue arrows denote physiological signalling. MTOC, microtubule-organizing centre. Schematic created with BioRender.com.

Formation of the DC–T cell IS requires the coordinated alignment of antigen presentation, co-stimulation, adhesion, cytoskeletal remodelling, membrane organization and localized metabolic support^27^ (Fig. 4). On the DC side, antigen uptake through phagocytosis is followed by lysosomal processing and ER-phagosome-associated antigen loading, allowing peptide–HLA complexes to be displayed at the contact interface^139^. In parallel, PRR- and TLR-induced PI3K–Akt–mTORC1 signalling supports DC activation, CD80/86 expression, cytoskeletal rearrangement and membrane fluidity, thereby enabling stable engagement with T cells through pMHC–TCR, CD80/CD86–CD28, and ICAM-1–LFA-1 interactions^140^.

Once antigen-bearing DCs contact cognate T cells, receptor engagement initiates spatially organized signalling at the synaptic interface. TCR stimulation activates proximal signalling through LCK, ZAP70 and LAT, which recruits PI3K and activates the downstream Atk–mTORC1 pathway^137^. This signalling cascade induces Myc- and HIF-1α-dependent metabolic remodelling, which supports T cell activation and effector differentiation^137,141^. At the same time, co-stimulatory signalling through CD28 reinforces PI3K–Akt–mTORC1 activity, linking antigen recognition to anabolic growth and effector programming^137^.

These early signalling events are physically organized within the supramolecular activation clusters (SMACs) of the IS. TCR signalling complexes accumulate towards the central SMACs, whereas LFA-1-mediated adhesion stabilizes the peripheral SMACs^142,143^. This spatial organization depends on rapid actin remodelling. ARP2/3, WASp and HS1 generate branched actin networks that support TCR microcluster formation and centripetal movement towards the central SMACs, whereas formin family proteins, including FMNL1 and mDia1, generate unbranched actin filaments that stabilize the IS and support microtubule-organizing centre (MTOC) polarization^144,145^. Because actin polymerization/depolymerization, receptor clustering and membrane spreading require continuous ATP hydrolysis, IS assembly represents a highly energy-demanding process during early T cell activation.

To meet these local energetic demands, the T cell rapidly polarizes its intracellular trafficking and bioenergetic machinery towards the synapse^138^. The MTOC relocates from the rear of the cell to the synaptic interface through dynein-driven microtubule capture shrinkage, a process supported by local glycolysis and hexokinase activity^146,147^. This MTOC polarization biases Golgi, ER and vesicle trafficking towards the IS, facilitating delivery of signalling components, metabolic cargoes such as glucose transporters and glycolytic enzymes, and secretory machinery required for polarized cytokine release^138,146^. In this way, the MTOC acts as a polarity hub that couples synaptic architecture to localized metabolic supply.

Mitochondria also rapidly translocate along the cytoskeleton towards the central SMACs, where they provide localized ATP/ADP gradients and buffer Ca^2+^ influx^29,138^. Their positioning near CRAC/ORAI channels allows mitochondria to take up incoming Ca^2+^, preventing Ca^2+^-dependent channel inactivation and sustaining the Ca^2+^ signals required for NFAT activation^148^. Kv1.3/KCa3.1 channels localized at the IS further help to maintain membrane potential and support CRAC-mediated Ca^2+^ entry^149^. Thus, mitochondrial polarization links local ATP supply, Ca^2+^ buffering and transcriptional activation at the synaptic microdomain.

In addition to mitochondrial support, the IS contains localized metabolic circuits that amplify signal transduction independently of the global glycolytic burst. Upon TCR engagement, the LCK-ZAP70 axis promotes TCR microcluster movement and is accompanied by increased intrasynaptic glucose-6-phosphate dehydrogenase (G6PD) activity and pentose phosphate pathway flux, generating NADPH and controlled ROS signals that reinforce proximal signalling^150^. Ionic and adhesion-dependent mechanisms further stabilize this platform; for example, Mg²⁺ binding to the metal ion-dependent adhesion site (MIDAS) of LFA-1 promotes its high-affinity interaction with ICAM-1, strengthening early DC–T cell adhesion and activation^151^.

Together, these spatially coordinated events establish the IS as a localized metabolic and signalling hub that couples antigen recognition to effector programming. This synapse-centred metabolic organization allows DC-derived TCR and co-stimulatory signals to be translated ultimately into PI3K-Akt-mTORC1 activation, polarized cytokine secretion and effector T cell differentiation^152^.

### Tumour microenvironment-derived disruption of synapse-specific metabolism

In the TME, tumour-derived metabolic stressors disrupt synapse-localized processes in ways that are distinct from global suppression of T cell activation^27^. Because the IS relies on localized ATP supply, Ca^2+^ influx, membrane organization and cytoskeletal remodelling, metabolic stress can destabilize productive DC–T cell communication even when physical contact is preserved.

Glucose deprivation represents a direct threat to this synapse-specific metabolic architecture. In DCs, glucose restriction attenuates the Akt–mTORC1 signalling and significantly reduces the expression of antigen-presenting and co-stimulatory molecules such as HLA, CD80 and CD86, thereby weakening the delivery of signal 1 and signal 2 at the DC–T cell interface^74,81^. In T cells, insufficient glucose availability limits the ATP required for actin polymerization, dynein-mediated MTOC docking and vesicle trafficking towards the IS^146,153^. In addition, glucose scarcity can alter N-linked glycosylation of surface receptors, including the TCR, thereby altering the glycocalyx and disrupting the close membrane apposition required for stable pHLA–TCR engagement^154,155^.

Lactate and acidosis further impair the synaptic microdomain by targeting antigen-processing compartments in DCs and Ca^2+^-dependent signalling in T cells. In DCs, tumour-derived lactate and acidosis can perturb lysosomal and endosomal antigen-processing pathways and reduce cross-presentation efficiency, at least in part by altering membrane trafficking, which weakens the supply of pHLA complexes available for synaptic engagement^86,87^. In T cells, high extracellular lactate can interfere with lactate/H^+^ handling across MCT transporters, promoting intracellular lactate accumulation and pH imbalance that weaken synapse–proximal signalling^156^. Reduced intracellular pH interferes with STIM1–ORAI1 coupling and CRAC channel activity, thereby collapsing TCR-induced Ca^2+^ flux at the synaptic microdomain and diminishing NFAT activation^157^.

In the TME, dying cancer cells release ATP, which functions as a danger signal. Extracellular ATP is rapidly dephosphorylated to AMP by CD39 and subsequently converted to adenosine by CD73, whereas extracellular adenosine levels are further regulated by adenosine deaminase (ADA)-mediated degradation and cellular uptake through nucleoside transporters^158^. In T cells, adenosine signals predominantly through the adenosine receptor subtype A_2A_ (A_2A_R), increasing intracellular cAMP and thereby suppressing T cell activation^159^. Mechanistically, adenosine-rich conditions functionally converge on the IS by suppressing membrane-proximal TCR signalling through the A_2A_R–cAMP–PKA axis, thereby reducing ZAP70 phosphorylation, Akt–ERK activation, Ca^2+^ influx and productive CD8^+^ T cell priming^160^.

Amino acid stress also converges on the structural and signalling machinery of the IS. For example, lLarginine depletion downregulates CD3ζ expression, a key component of the TCR signalling complex, and impairs cofilin dephosphorylation required for actin cytoskeletal remodelling during synapse formation^161,162^. Consequently, T cells fail to efficiently propagate TCR signals from the synaptic interface. In addition, glutamine deprivation reduces the intracellular pool of UDP–GlcNAc and leads to decreased O-GlcNAcylation of signalling proteins such as T cell receptor and transcription factors such as c-Myc, thereby preventing sustained T cell activation signalling and metabolic reprogramming^163^.

Lipid dysregulation imposes an additional layer of synapse-specific disruption by altering membrane architecture and signalosome stability. In DCs, excessive lipid accumulation in lipid bodies can impair antigen trafficking and cross-presentation, despite preserved MHC class I expression, ultimately compromising pMHC display at the DC–T cell interface^11^. Oxidized lipids and ceramide species further perturb lipid-raft organization and TLR-dependent activation, reducing immunostimulatory cytokine secretion and DC priming capacity^104^. In T cells, lipid rafts serve as essential platforms for TCR signalosome assembly by concentrating LCK, LAT, and other proximal signalling molecules^164,165^. At the membrane level, ACAT1-mediated esterification of free cholesterol into lipid droplets reduces plasma membrane cholesterol, destabilizes lipid-raft formation and compromises the physical and functional platform required for efficient TCR signalling^166,167^. Tumour-derived lipid mediators, including oxysterols, oxidized phospholipids and lysophosphatidic acid, can further disrupt TCR microcluster coalescence, LFA-1 avidity and actin-dependent synapse stabilization^165,168,169^. Ceramide accumulation further inhibits PKCθ phosphorylation, thereby impairing the PKCθ–NF-κB axis that links TCR/CD28 engagement to transcriptional activation^104^.

Collectively, these TME-derived metabolic perturbations establish the IS as a vulnerable metabolic checkpoint within the CIC. By selectively disrupting synapse-localized bioenergetic supply, membrane organization, cytoskeletal coordination and signal propagation, the TME weakens the ability of DCs and T cells to convert antigen recognition into sustained activation. Thus, metabolic stress at the IS can redirect otherwise productive DC–T cell encounters towards inefficient, tolerogenic or dysfunctional outcomes.

## Therapeutic strategies targeting immunometabolic mechanisms

### Integrating metabolic vulnerabilities into precision oncology

Metabolic reprogramming provides a framework for identifying tumour-specific vulnerabilities across carbohydrate, amino acid and lipid metabolic pathways, underpinning precision oncology strategies that exploit cancer-selective metabolic dependencies while sparing normal cellular functions^170,171^. However, the therapeutic relevance of metabolic intervention extends beyond direct tumour cytotoxicity. By reshaping nutrient availability, redox balance, lipid composition and immunosuppressive metabolite accumulation within the TME, metabolism-targeted therapies may also influence DC priming, DC–T cell synapse formation, T cell trafficking and effector function^1,89,172^. Importantly, not all metabolic inhibitors discussed below have established direct immune-modulatory activity in patients. Therefore, we distinguish between agents that primarily induce tumour-intrinsic metabolic stress, agents with demonstrated or plausible effects on immune or stromal compartments and agents whose immune consequences remain context dependent or inferred from preclinical and correlative clinical data. In this framework, the CIC stages indicated in Table 2 should be interpreted as the dominant immune processes most likely to be affected by each intervention, rather than as exclusive or fully validated sites of action.

**Table 2:** Therapeutic strategies targeting metabolic checkpoints across the cancer-immunity cycle (CIC).

Target	Modality	Primary CIC positioning	Primary target compartment	Mechanism	Cancer type	ClinicalTrials. gov ID	Status
GLUT1 transporter	BAY-876	Step 1; potentially Steps 6–7	Tumour cell; indirect immune effect	Inhibits glucose uptake via GLUT1 blockade; triggers severe glycolytic insufficiency and may promote ICD or antigen release	Colorectal, hepatocellular	NCT04674306	I
Hexokinase 2	2-Deoxy-D-glucose (2-DG)	Step 1; potentially Steps 2, 6–7	Tumour cell; DC–T cell context-dependent	Competitive glucose analogue; blocks glycolysis; starves tumour cells of energy and promotes ICD	Pancreatic, glioblastoma	NCT00096707	I
PDH and α-KGDH	CPI-613 (devimistat)	Step 1; potentially Steps 6–7	Tumour mitochondria; inferred immune effect	Disrupts TCA cycle enzymes; induces metabolic crisis; may increase antigen release through tumour metabolic crisis	Biliary tract cancer	NCT04203160	I
Arginine deiminase	ADI-PEG20 + cisplatin	Step 1; potentially Steps 3, 6–7	ASS1-deficient tumour; arginine milieu	Depletes extracellular arginine; starves tumour cells; triggers tumour necrosis	Metastatic melanoma, advanced solid tumour	NCT01219348	I/II
Cysteine/glutathione depletion	Imexon	Step 1; potentially Steps 2, 6–7	Tumour redox metabolism; TME milieu	Thiol oxidation; depletes GSH, increases ROS; induces oxidative stress, potentially increasing tumour antigenicity	Advanced solid tumours, B cell non-Hodgkin lymphoma	NCT01314014	II
xCT/cysteine transporter	Sulfasalazine	Step 1; potentially Steps 2, 6–7	Tumour redox metabolism; immune-context dependent	xCT inhibition; blocks cystine uptake, inhibits GSH synthesis; induces ferroptosis and enhances tumour antigenicity	Glioblastoma	NCT04205357	I/II
SCD1	MTI-301 (SSI-4)	Step 1; potentially Steps 6–7	Tumour lipid metabolism; inferred immune effect	Blocks MUFA synthesis; induces ferroptosis	HCC, CCA, advanced solid tumours	NCT06911008	Not yet recruiting
PPAR-α	Fenofibrate	Step 1; potentially Steps 6–7	Tumour lipid/FAO metabolism	Inhibits PPAR-α-mediated transcription of fatty acid oxidation genes	Invasive cervical cancer	NCT06191133	I
FASN	TVB-2640	Step 1; potentially Steps 2–3	Tumour lipogenesis; DC lipid stress indirect	Inhibits fatty acid synthesis; reduces lipid pools; may relieve lipid-mediated DC dysfunction; induces metabolic stress and ICD	Breast, CRC, HCC	NCT02223247	I
PI3Kα/SREBP	Alpelisib (BYL719)	Step 1; potentially Steps 2–3	Tumour PI3K–lipogenesis axis	Indirectly inhibits SREBP; reduces lipogenesis; may improve antigen presentation quality	Breast cancer	NCT01493843	I/II
Arginase 1/2	INCB001158	Steps 2–3; potentially Steps 6–7	Myeloid/MDSCs; T cells indirect	Restores arginine; reverses myeloid-mediated immunosuppression; may support co-stimulatory molecule expression (CD80/86)	HNSCC, CRC, HCC	NCT02903914	I
Arginase	Navoximod (CB-1158)	Steps 2–3; potentially Steps 6–7	Myeloid/MDSCs; DC–T cells indirect	Inhibits arginase; restores the metabolic fitness of DCs to prevent tolerogenic phenotype	HCC	NCT02903914	I
IDO1	Epacadostat + pembrolizumab	Steps 2–3; potentially Steps 6–7	Tumour/myeloid IDO1; T cells indirect	Blocks tryptophan→kynurenine; maintains DC activation and priming capacity	Melanoma, sarcoma	NCT03414229	II
IDO pathway	Indoximod (1-MT)	Steps 2–3; potentially Steps 6–7	Trp-kynurenine milieu; T cells indirect	Competes with tryptophan sustains antigen-priming capacity	Breast cancer	NCT01792050	II
GLS	CB-839 (telaglenastat)	Step 3; potentially Steps 6–7	Tumour glutamine metabolism; T cells indirect	Inhibits glutamine→glutamate; modulates glutamine competition and tumour glutamine dependence	RCC, NSCLC, mesothelioma	NCT02071862	I
SOAT1	Nevanimibe (ATR-101)	Step 3; potentially Steps 6–7	Tumour sterol metabolism; T cell effect inferred	Inhibits cholesterol esterification; alters membranes; may support lipid raft-dependent TCR clustering	ACC	NCT01898715	I
CD73	Oleclumab + osimertinib	Step 3; potentially Steps 6–7	Tumour/stromal/immune CD73; T cells indirect	Inhibits CD73-mediated adenosine production; prevents adenosine-induced suppression of TCR signalling	EGFR-mutated NSCLC	NCT03381274	I/II
BTK	PCI-32765 (ibrutinib)	Steps 6–7; potentially Step 3	B cell/myeloid/T cell signalling	BTK inhibitor; polarized F-actin synapse between T cells and tumour	CLL, SLL, PLL	NCT01589302	II
PDK	DCA	Steps 6–7; potentially Step 3	Tumour mitochondria; TIL fitness indirect	Activates PDH; shifts metabolism towards OXPHOS in cancer; sensitizes cancer to cytotoxic T cells	GBM, lung	NCT01111097	I/II
Mitochondrial complex I	Sapanisertib + metformin	Steps 6–7	Tumour and immune mitochondria	mTORC1/2 + complex I inhibitor; dual metabolic stress; may enhance CD8^+^ cytotoxic function in selected contexts	Advanced solid tumours	NCT03017833	I
MCT1 transporter	AZD3965	Steps 6–7; potentially Step 3	Tumour lactate transport; T cells indirect	Blocks lactate export; intracellular acidification; may reduce lactate-associated T cell suppression	Advanced solid tumours, lymphoma	NCT01791595	I
COX-2	Celecoxib	Steps 6–7; potentially Steps 2–3	Tumour/myeloid PGE_2_ axis; T cells indirect	COX-2 inhibitor; inhibits PGE₂ production; blocks MDSC recruitment; relieves T cell suppression	HCC	NCT07174570	II recruiting

*ACC* adrenocortical carcinoma, *CCA* cholangiocarcinoma, *CLL* chronic lymphocytic leukaemia, *CRC* colorectal cancer, *DC* dendritic cell, *DCA* dichloroacetate, *FAO* fatty acid oxidation, *GBM* glioblastoma multiforme, *GSH* glutathione, *HCC* hepatocellular carcinoma, *HNSCC* head and neck squamous cell carcinoma, *ICD* immunogenic cell death, *MDSC* myeloid-derived suppressor cells, *MUFA* monounsaturated fatty acid, *NSCLC* non-small-cell lung cancer, *PLL* prolymphocytic leukaemia, *RCC* renal-cell carcinoma, *ROS* reactive oxygen species, *SLL* small lymphocytic lymphoma, *TCA* tricarboxylic acid, *TME* tumour microenvironmen.

### Tumour-intrinsic metabolic targeting: inducing metabolic crisis and potential immunogenic antigen release

A first group of metabolic therapies primarily targets tumour-intrinsic metabolic dependencies. These agents are most relevant to the antigen-release phase of the CIC, because metabolic collapse, oxidative stress, ferroptosis or mitochondrial dysfunction may increase tumour cell death and potentially enhance antigen availability. However, for many of these agents, immune activation remains indirect or incompletely established.

In highly glycolytic tumours, inhibition of glucose metabolism can expose a strong metabolic liability. The GLUT1 inhibitor BAY-876 restricts glucose uptake and may trigger glycolytic insufficiency in GLUT1-dependent tumours^173,174^. Although such inhibition may theoretically reduce lactate-mediated immune suppression and improve T cell function, its clinical immune-modulatory effect should be regarded as indirect unless supported by immune biomarker data. Similarly, 2-Deoxy-D-glucose (2-DG), a glucose analogue that interferes with glycolysis through hexokinase-dependent trapping, has been evaluated in combination with docetaxel and demonstrated clinical tolerability^175^. Its dominant activity is tumour-directed energetic stress, although immune effects may arise secondarily through increased tumour-cell death, altered antigen release, or changes in the glucose–lactate balance of the TME.

Mitochondrial metabolic targeting provides another route to tumour-selective metabolic crisis. CPI-613 (devimistat), which targets TCA-cycle-associated enzymes including pyruvate dehydrogenase (PDH) and α-ketoglutarate dehydrogenase (α-KGDH), has shown clinical activity in combination regimens for biliary tract cancer^176^. In the CIC framework, this strategy is most appropriately positioned at antigen release and potential immunogenic cell death. Nevertheless, any downstream expansion of effector T cells or immune activation should be presented as correlative or inferred, because the primary pharmacological target is tumour mitochondrial metabolism rather than immune cells.

Amino acid and redox-targeting strategies similarly exploit tumour-specific metabolic dependencies. ADI-PEG20 depletes extracellular arginine and selectively affects ASS1-deficient tumours^177^. Although arginine depletion can influence T cell biology, ADI-PEG20 is best described as a tumour-directed arginine starvation strategy, with immune consequences that depend on tumour ASS1 status, host arginine metabolism and the balance between tumour killing and possible T cell nutrient limitation. When combined with cisplatin, ADI-PEG20 showed stable disease in 41% of patients, consistent with the association between low ASS1 expression, advanced disease stage and heightened sensitivity to arginine-depleting therapies in ovarian and other cancers^177^. Redox-directed agents such as imexon and sulfasalazine further illustrate this point. Imexon depletes glutathione and increases ROS, whereas sulfasalazine inhibits xCT-mediated cystine uptake and reduces glutathione synthesis^178–180^. These interventions may increase oxidative tumour stress, ferroptotic susceptibility and antigenicity, but their effects on DCs and T cells are likely context dependent because immune cells also require cysteine/cystine and redox buffering for proliferation and effector function. Thus, these agents should be framed as tumour redox modulators with potential, rather than established, immune-enhancing activity.

Lipid metabolic interventions also fall partly into this tumour-intrinsic category. MTI-301, an SCD1 inhibitor, is designed to reduce monounsaturated fatty acid pools and promote lipid peroxide accumulation, thereby inducing ferroptosis^89^. Fenofibrate, a PPARα agonist used clinically for dyslipidaemia, has been reported to induce cell-cycle arrest and apoptosis in cancer models and is being evaluated in cervical neoplasia and cervical cancer^181^. For both agents, the most defensible link to the CIC is through altered tumour lipid metabolism, ferroptotic stress or tumour-cell death, whereas immune modulation should be described as potential or inferred until immune-specific clinical evidence becomes available.

### Reprogramming suppressive nutrient and lipid milieus to support dendritic cell priming and T cell activation

A second group of therapies more directly targets the metabolic crosstalk between tumour cells, myeloid cells, DCs and T cells. These interventions are particularly relevant to antigen processing and presentation, DC priming and early T cell activation, because they modulate the availability of immunologically critical amino acids or reduce suppressive metabolites in the TME.

Arginine metabolism represents one of the clearest examples of immune-metabolic crosstalk. Arginase activity in myeloid-derived suppressor cells and other suppressive myeloid populations depletes extracellular arginine, impairing TCR signalling, CD3ζ expression, T cell proliferation and effector function^182^. The arginase inhibitor INCB001158 is therefore more directly aligned with immune restoration than tumour-directed arginine depletion strategies. In combination with pembrolizumab, INCB001158 increased plasma arginine levels and was associated with enhanced CD8⁺ T cell infiltration in patients with PD-1/PD-L1-naïve squamous cell carcinoma of the head and neck^183^. Navoximod, another arginase inhibitor, follows a similar rationale by relieving myeloid-mediated arginine restriction and supporting T cell and DC metabolic fitness^182^. These agents should be emphasized as examples in which the therapeutic target resides primarily in the myeloid/TME compartment rather than exclusively in cancer cells.

Tryptophan catabolism through the IDO/TDO–kynurenine–AhR axis is another major immune-metabolic checkpoint. IDO1 inhibition with epacadostat is aimed at reducing kynurenine accumulation, preserving tryptophan availability and preventing tolerogenic immune programming^184^. Although the clinical efficacy of IDO1 inhibitors has been variable across trials, their mechanistic connection to DC priming, T cell activation and immune suppression is well established. Indoximod, a tryptophan mimetic, acts differently by counteracting tryptophan-starvation signalling and sustaining mTORC1-dependent immune-cell survival and function^185^. These agents should therefore be positioned primarily at CIC Steps 2–3, where antigen presentation and productive T cell priming are metabolically vulnerable.

Lipid metabolism also shapes DC function and antigen presentation. Tumour-driven lipid accumulation, oxidized phospholipids and altered fatty acid synthesis can impair DC antigen handling and promote tolerogenic myeloid states. The FASN inhibitor TVB-2640 reduces de novo lipogenesis and has shown target engagement with manageable safety in early clinical studies^186^. Its immune relevance is best framed as indirect but mechanistically plausible, because reducing tumour lipid output or lipid stress may relieve DC dysfunction. Similarly, alpelisib, a PI3Kα-selective inhibitor, suppresses PI3K–SREBP-driven lipogenic programs in PIK3CA-mutant breast cancer^187^. Although its primary indication is tumour-intrinsic PI3K pathway inhibition, reduced lipogenic signalling may secondarily influence antigen presentation quality and lipid-mediated immune suppression. Thus, these lipid-directed agents connect to CIC Steps 2–3 most strongly when the tumour lipid program contributes to DC dysfunction or defective priming.

### Restoring T cell fitness, synapse competence, and effector function in the tumour bed

A third therapeutic category targets metabolic barriers that interfere with T cell activation, immunological synapse formation and tumour-cell killing. These interventions are most relevant to CIC Steps 3 and 6–7, where T cells must maintain mitochondrial fitness, ionic balance, cytoskeletal remodelling and cytotoxic activity within a metabolically hostile TME.

Glutamine metabolism illustrates the difficulty of separating tumour-intrinsic and immune-directed effects. Telaglenastat (CB-839), a glutaminase inhibitor, suppresses tumour glutamine metabolism and has been evaluated in renal-cell carcinoma, NSCLC, pancreatic cancer, mesothelioma and lymphoma^188^. Because activated T cells also use glutamine for biosynthesis and signalling, the immune effect of glutaminase inhibition is likely dose, context and combination dependent. In tumours with glutamine addiction, tumour-directed glutamine blockade may relieve metabolic competition or remodel the TME, but direct enhancement of T cell function should be stated cautiously unless supported by immune profiling.

Membrane lipid composition is also relevant to immunological synapse competence. Nevanimibe (ATR-101), an ACAT1/SOAT1 inhibitor, decreases cholesterol esterification and can alter free cholesterol availability^189^. As cholesterol-rich membrane domains contribute to TCR clustering and synapse organization, ACAT1 inhibition provides a mechanistic rationale for improving T cell synapse stability^167^. However, because clinical evidence for direct T cell synapse enhancement remains limited, this link should be described as inferred from membrane biology and preclinical immune studies rather than as an established clinical effect.

Adenosine metabolism represents a more direct immune-suppressive axis at the tumour–T cell interface. CD73-mediated conversion of AMP to adenosine suppresses TCR signalling, cytokine production, cytotoxicity and T cell proliferation^190^. Oleclumab, an anti-CD73 antibody, therefore, directly targets an immunosuppressive metabolic pathway. In EGFR-mutant NSCLC, oleclumab combined with osimertinib showed limited objective responses but suggested potential benefit in progression-free survival compared with historical expectations^191^. This strategy is best positioned at the DC–T cell synapse and effector phases, where adenosine restrains T cell activation and cytotoxic function.

Several agents originally developed for non-metabolic oncogenic or inflammatory targets may also influence immune-metabolic fitness. Ibrutinib, a BTK inhibitor, can alter B cell, myeloid and T cell signalling, reduce PD-1/PD-L1-associated immune suppression, promote Th1-skewed responses and enhance actin polarization required for cytotoxic synapse formation^192–194^. Although this agent is not a canonical metabolic inhibitor, it is relevant here because synapse formation and cytotoxic function depend on the coordinated integration of signalling events, cytoskeletal remodelling and metabolic support. Likewise, celecoxib inhibits COX-2-dependent PGE_2_ production, thereby reducing a lipid-derived suppressive mediator that limits DC function, promotes suppressive myeloid recruitment and impairs CD8⁺ T cell effector activity^195^. Compared with many tumour-intrinsic metabolic inhibitors, COX-2 blockade has a clearer connection to immune suppression through the PGE_2_ axis.

Finally, lactate and mitochondrial-targeting therapies may improve the effector phase of the CIC by modifying the metabolic conditions encountered by tumour-infiltrating lymphocytes. AZD3965 inhibits MCT1-mediated lactate transport, leading to intracellular acidification and metabolic stress in MCT1-dependent tumours^196^. Its potential immune benefit lies in reducing lactate-driven suppression or altering tumour acidity, but direct enhancement of T cell killing remains an indirect and context-dependent possibility. Metformin, particularly in combination strategies such as sapanisertib plus metformin, affects mitochondrial complex I and mTOR signalling^197,198^. These interventions may influence both tumour metabolism and T cell mitochondrial fitness, including memory differentiation and cytotoxic capacity. Dichloroacetate (DCA), a PDK inhibitor, promotes pyruvate entry into mitochondrial oxidation and has been investigated in glioblastoma and lung cancer^199^. In the immune context, shifting mitochondrial metabolism may help to relieve oxidative exhaustion or improve effector function, but this should be interpreted as context-dependent because the same metabolic pathways are shared by tumour and immune cells.

### Metabolic engineering of cell-based immunotherapies

Therapeutic strategies targeting cancer metabolic reprogramming have evolved from simply disrupting tumour-cell metabolic dependencies towards integrated approaches that also metabolically arm adoptive cell therapies and enhance their persistence within the TME^200^.

In CAR-T cells, metabolic gene modulation critically influences cellular persistence, differentiation trajectories and cytotoxic capabilities^114,200^. Overexpression of fumarase or PRODH2 increases mitochondrial mass and enhances OXPHOS, promoting memory formation and sustained cytokine production^200,201^. Loss of Regnase-1 upregulates TCF1 expression, facilitating long-lived memory phenotypes, improving CAR-T cell durability and antitumour activity. Overexpression of nutrient transporters in the SLC family, such as SLC7A5, SLC7A11 and GLUT1, can support CAR-T cell proliferation and functional competence under nutrient-deprived, lactate-rich conditions in the TME^114,200,201^. Similarly, engineering CAR-T cells to express metabolic enzymes that restore nutrient fitness or catabolize immunosuppressive metabolites, such as kynureninase, can reduce exhaustion and reinforce memory programming^200,201^.

Beyond genetic reprogramming, cytokine-based metabolic modulation has also been explored. Administration of IL-10–Fc recombinant protein induced a metabolic switch towards pyruvate- and mitochondrial pyruvate carrier (MPC)-dependent OXPHOS, reinvigorating terminally exhausted CD8⁺ T cells to reacquire effector phenotypes and improving antitumour activity^202^. These findings support the broader concept that metabolically reprogramming T cells can enhance persistence and function in metabolically hostile tumours. Consistent with this rationale, metabolically armed BCMA-directed CAR-T cells for multiple myeloma are currently undergoing a phase I clinical trial (NCT07085559).

DC-based vaccines can likewise be metabolically tuned. For example, a DC-vaccine study using the metabolic glycan label Ac4ManNAz showed that glycoengineering DCs reduced membrane mobility while enhancing CD86 expression and CD8⁺ T cell priming in mouse BMDCs and the E.G7-OVA tumour model^203^. These results support the notion that fine-tuning membrane composition and metabolic pathways in DCs can improve synapse quality and T cell activation.

Metabolic reprogramming in cell therapy continues to advance through genetic engineering, targeted modulation of metabolic pathways, pharmacological co-treatments, and innovations in manufacturing. Rather than relying solely on direct cytotoxic mechanisms, these approaches are aimed at reconfiguring intracellular metabolic networks and enhancing adaptation to the TME. In the context of the CIC, combining tumour-directed metabolic inhibitors with immunometabolically optimized DC- and T cell-based therapies may help to overcome metabolic checkpoints at both the DC–T cell and T cell–tumour synapses. However, the strength of evidence varies substantially across agents and platforms. Therefore, integrating metabolic therapy into precision oncology will require biomarkers that define not only tumour metabolic dependencies but also the immune-cell compartment most likely to benefit from metabolic rewiring.

## Future directions and translational outlook

Over the past decade, it has become evident that cancer does not rely on a single metabolic program but instead occupies a heterogenous spectrum of metabolic states that support tumour growth and immune evasion. This diversity provides critical insights into tumour-growth dynamics and prognostic implications. Some tumours adopt a high-glycolysis, “sprint-like” strategy — characterized by rapid but inefficient ATP generation — that favours explosive proliferation, high lactate output and aggressive behaviour, often correlating with poor prognosis. Conversely, others harness enhanced lipid metabolism and TCA-cycle-driven OXPHOS, representing a more differentiated, “marathon-like” strategy that supports long-term survival, stress resistance and metastatic seeding. This conceptual framework emphasizes that the type of metabolic program, rather than just metabolic rate, dictates tumour aggressiveness and clinical outcomes. Consequently, future research must move beyond broad metabolic inhibition to targeting the specific bioenergetic nodes that drive these distinct survival strategies.

### High-resolution profiling and real-time imaging

To unravel this metabolic heterogeneity, modern translational immunology is increasingly relying on enabling platforms that bridge basic discovery and clinical application. Single-cell multi-omics — encompassing single-cell RNA sequencing, assay for transposase-accessible chromatin with sequencing and cellular indexing of transcriptomes and epitopes by sequencing — now provides high-resolution profiling of transcriptomic, epigenetic and protein states, revealing the metabolic and functional diversity of immune and stromal cells within the TME^110^. Complementing this, in situ metabolic mapping and metabolite profiling (e.g. ^13^C-glucose infusion) allow for the visualization of dynamic metabolic fluxes, capturing bioenergetic shifts in CD8⁺ T cells during infection or cancer at near single-cell resolution^110,204^. Furthermore, advances in bioimaging now permit the real-time, non-invasive analysis of metabolic dynamics^204,205^. Label-free two-photon fluorescence lifetime microscopy tracks intracellular NAD(P)H dynamics, enabling researchers to quantify single-cell shifts between OXPHOS and glycolysis in vivo and ex vivo. Notably, this method has revealed that TCR stimulation induces a rapid glycolytic switch within minutes, with metabolic co-factors concentrating specifically at the IS^205^. Such tools now make it feasible to directly visualize metabolic checkpoint engagement at the DC–T and T–tumour synapses in living systems.

### Next-generation disease modelling: from 2D to microphysiological systems

To translate these findings into therapeutic success, experimental systems must faithfully reproduce the physical and chemical constraints of the TME. Microfluidics-based microphysiological systems are advancing rapidly to model complex tumour–immune interactions in dynamic 3D environments^206^. Unlike traditional, static 2D cultures, these systems recapitulate TME architecture, oxygen/nutrient gradients, fluid pressure, and extracellular matrix–cell interactions^206^. A notable example is the “lymph node-on-a-chip”, which recreates key structural, stromal and fluidic features of human lymphoid tissue^207^. Such platforms are particularly useful for studying metabolic–immunological mechanisms because they can impose and monitor spatial gradients of oxygen, glucose, lactate, amino acids, pH and cytokines while simultaneously tracking DC migration, antigen presentation, T cell priming, proliferation and effector differentiation^206,208^. By co-culturing DCs and T cells on a 3D fibroblastic reticular cell network, lymph node-on-a-chip systems enable real-time visualization of immune-cell positioning, CCR7-dependent migration and early antigen-priming events under defined metabolic constraints^207^. They may also be adapted to model metabolic stress conditions relevant to lymph-node metastasis, thereby enabling the discovery of metabolic checkpoints that disrupt early tumour-specific immune priming and the screening of personalized immunometabolic therapies^208^. The integration of tumour organoids, 3D extracellular matrices and microfluidic TME chips will further facilitate high-throughput drug screening and validation of metabolic–immunological mechanisms, helping to bridge the gap between conventional preclinical models and human physiology.

### Towards precision metabolic immunotherapy

The ultimate goal of these technological advancements is the realization of personalized metabolic immunotherapy. Advanced multimodal platforms are beginning to support patient stratification based on metabolic biomarkers; for instance, distinct metabolic profiles in patients with NSCLC have been shown to correlate with enhanced sensitivity to PD-1 therapy^209^. Moving forward, a key priority is the construction of metabolic–immune spatial atlases that integrate tumour metabolic phenotypes, immune-cell states (e.g. stimulatory versus tolerogenic DCs), and synapse-quality metrics^110,210^. These atlases will enable the stratification of patients by dominant metabolic bottlenecks and predict responsiveness to immunometabolic interventions^89,110^. Trial designs that specifically target the DC–T cell metabolic interface — preserving synaptic bioenergetics while disrupting tumour metabolic defences — offer the most promising avenue for next-generation therapies^27,89,210^. Such interface-focused trials will be particularly important for therapies directed at the DC–T or tumour–T metabolic axis — including DC vaccines, costimulatory agonists, bispecific T cell engagers and metabolically engineered CAR or TCR-T cells — where success depends on a favourable metabolic context at both priming and effector sites.

Ultimately, metabolically informed, synapse-aware precision immunotherapy is aimed not only at attacking tumour cells but at restoring and stabilizing the DC–T cell axis at the centre of antitumour immunity. By integrating metabolic targeting with sophisticated enabling platforms and carefully designed clinical trials, it may become possible to convert transient responses into durable, long-term tumour control for a broader range of patients.
